# Safety and Immunogenicity of a Second Dose of an Investigational Maternal Trivalent Group B Streptococcus Vaccine in Nonpregnant Women 4–6 Years After a First Dose: Results From a Phase 2 Trial

**DOI:** 10.1093/cid/ciz737

**Published:** 2019-08-08

**Authors:** Geert Leroux-Roels, Zourab Bebia, Cathy Maes, Annelies Aerssens, Fien De Boever, Luca Grassano, Giada Buffi, Immaculada Margarit, Annette Karsten, Stephen Cho, Karen Slobod, Bartholomew Corsaro, Ouzama Henry

**Affiliations:** 1 Center for Vaccinology, Ghent University and Ghent University Hospital, Belgium; 2 GlaxoSmithKline (GSK), Rockville, Maryland; 3 GSK, Siena, Italy; 4 GSK, Marburg, Germany; 5 Novartis, Cambridge, Massachusetts; 6 GSK, Cambridge, Massachusetts

**Keywords:** group B streptococcus, maternal immunization, second dose, safety, immunogenicity

## Abstract

**Background:**

Maternal immunization against group B streptococcus (GBS) could protect infants from invasive GBS disease. Additional doses in subsequent pregnancies may be needed. We evaluated the safety and immunogenicity of a second dose of an investigational trivalent CRM_197_-glycoconjugate GBS vaccine (targeting serotypes Ia/Ib/III), administered to nonpregnant women 4–6 years postdose 1.

**Methods:**

Healthy women either previously vaccinated with 1 dose of trivalent GBS vaccine 4–6 years before enrollment (n = 53) or never GBS vaccinated (n = 27) received a single trivalent GBS vaccine injection. Adverse events (AEs) were recorded. Serotype-specific (Ia/Ib/III) anti-GBS antibodies were measured by multiplex immunoassay prevaccination and 30/60 days postvaccination.

**Results:**

AEs were reported with similar rates after a first or second dose; none were serious. Of previously GBS-vaccinated women, 92%–98% had anti-GBS concentrations that exceeded an arbitrary threshold (8 µg/mL) for each serotype 60 days postdose 2 vs 36%–56% postdose 1 in previously non–GBS-vaccinated women. Of previously GBS-vaccinated women with undetectable baseline (predose 1) anti-GBS levels, 90%–98% reached this threshold postdose 2. For each serotype, anti-GBS geometric mean concentrations (GMCs) 30/60 days postdose 2 in previously GBS-vaccinated women were ≥200-fold higher than baseline GMCs. Among women with undetectable baseline anti-GBS levels, postdose 2 GMCs in previously GBS-vaccinated women exceeded postdose 1 GMCs in previously non–GBS-vaccinated women (≥7-fold).

**Conclusions:**

A second trivalent GBS vaccine dose administered 4–6 years postdose 1 was immunogenic with a favorable safety profile. Women with undetectable preexisting anti-GBS concentrations may benefit from a sufficiently spaced second vaccine dose.

**Clinical Trials Registration:**

NCT02690181

## Teaser

A second dose of the investigational trivalent group B streptococcus (GBS) vaccine, given 4–6 years after the first dose, elicited a robust immune response for each vaccine serotype in nonpregnant women, including in those with undetectable predose 1 anti-GBS antibody levels.

**(See the Brief Report by Crowell et al on pages 2706–9.)**

Group B streptococcus (GBS) is a leading cause of sepsis and meningitis in newborns and young infants, with an incidence of 0.49/1000 live births [1, 2]. Each year, more than 300 000 infants younger than 3 months are estimated to develop invasive GBS disease worldwide, resulting in 90 000 infant deaths [3]. Furthermore, an estimated 1%–4% of stillbirths are associated with GBS [4], and there is evidence of a possible link with preterm births [5]. GBS can be transmitted vertically from the mother’s rectovaginal tract to the fetus during pregnancy or parturition, making maternal colonization a major risk factor for infant GBS disease [6]. An estimated 11%–35% of pregnant women are colonized with GBS (totaling 21.7 million women) [3, 7].

Intrapartum antibiotic prophylaxis (IAP) in GBS-colonized pregnant women has substantially reduced the incidence of early-onset disease (onset during the first 7 days of life) [6, 8–10]. However, IAP has not reduced the rate of late-onset disease (onset between 7 and 90 days), does not prevent GBS-associated preterm or stillbirths, and its implementation is logistically challenging in low- and middle-income countries [6, 8, 10]. An effective prophylactic vaccine administered during pregnancy could complement IAP [6, 11–13]. For several GBS serotypes, an inverse relation has been shown between antibody levels against the capsular polysaccharide (CPS) in pregnant women and the risk of invasive GBS disease in their infants [6, 14–19]. This led to the use of serotype-specific GBS CPS for vaccine development [6, 11, 12]. An investigational trivalent vaccine that contains CPS from GBS serotypes Ia, Ib, and III, which together cause >85% of infant invasive GBS disease [2], conjugated to the CRM_197_ carrier protein (nontoxic mutant of diphtheria toxin) was well tolerated and immunogenic in nonpregnant and pregnant women [20–23]. Studies have also shown that anti-CPS antibodies induced by maternal immunization with this vaccine were transferred transplacentally to infants and persisted through a minimum of 3 months of age [20, 21, 23, 24].

The World Health Organization has articulated a preference for a single-dose maternal regimen, while acknowledging that a 2-dose regimen may need to be considered and that additional doses in subsequent pregnancies should be investigated [13]. The current study (an extension of a previous trivalent GBS vaccine trial in nonpregnant women [22]) was conducted to evaluate the safety and immunogenicity of a second dose of the trivalent GBS vaccine administered 4–6 years after the initial dose.

## METHODS

### Study Design, Participants, and Vaccines

**Figure 1.**
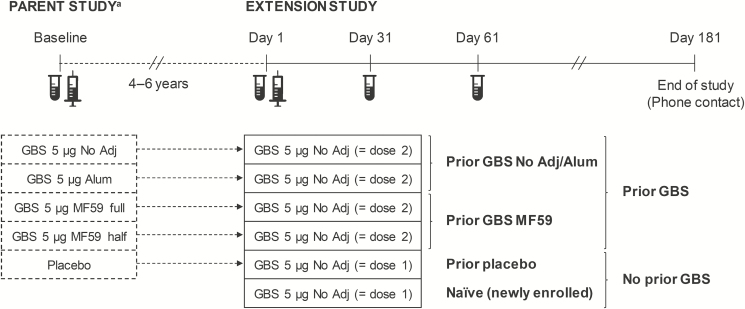
Study design. Abbreviations: alum, aluminum hydroxide-adjuvanted formulation; GBS, Group B streptococcus; MF59 full, full-dosage MF59-adjuvanted formulation; MF59 half, half-dosage MF59-adjuvanted formulation (a full dosage of MF59 contained 9.75 mg squalene, 1.18 mg polysorbate 80, 1.18 mg sorbitan trioleate, 0.66 mg sodium citrate dehydrate, and 0.04 mg citric acid monohydrate); no adj, nonadjuvanted formulation. ^a^For the parent study, only interventions relevant to the current analyses are presented. Vials indicate blood sampling for immunogenicity assessment. Syringes indicate vaccination in the parent or extension study. Braces indicate how the groups are pooled.

In a previous study (NCT01150123; parent study) aimed at selecting trivalent GBS vaccine formulations and schedules for potential use in pregnant and nonpregnant women, nonpregnant women were randomized to receive 1 or 2 injections (1 month apart) of 2 vaccine antigen dosages (5 or 20 µg), either nonadjuvanted, aluminum hydroxide (alum)-adjuvanted or adjuvanted with MF59 (half- or full-dosage; Figure 1) [22, 25]. The study included a placebo group as the comparator.

The current phase 2 extension study was a nonrandomized, controlled, open-label study with 6 parallel groups. Healthy, nonpregnant women aged 22–46 years who had received a single injection of any of the 5-µg formulations (nonadjuvanted, alum-adjuvanted, half- or full-dosage MF59-adjuvanted) or placebo 4–6 years earlier in the parent study were enrolled. To ensure an adequate number of controls, women who were not part of the parent study and never received a GBS vaccine (naïve group) were also enrolled (Figure 1). Inclusion and exclusion criteria are detailed in the Supplementary Methods.

Women in each of the 6 groups received a single injection of nonadjuvanted trivalent GBS vaccine containing CRM_197_-conjugated GBS CPS of serotypes Ia, Ib, and III (5 µg each). This constituted a second dose for 4 groups (all previously GBS-vaccinated in the parent study: prior GBS groups) and a first dose for the placebo group from the parent study (prior placebo group) and the newly enrolled naïve group (Figure 1). The vaccine was administered intramuscularly in the deltoid muscle of the nondominant arm.

The study was conducted at the Center for Vaccinology, Ghent University Hospital, Belgium, between March 2016 and November 2016 according to the principles of Good Clinical Practice, the Declaration of Helsinki, and applicable regulations. The Ghent University Hospital Commission for Medical Ethics approved the protocol and informed consent form. Each participant provided written informed consent before enrollment.

### Reactogenicity and Safety Assessment

Women received diary cards to record solicited adverse events (AEs) that occurred on days 1–7. Unsolicited AEs that occurred on days 1–31 were also recorded. On days 15, 121, and 181 (study end), participants received safety follow-up calls. The intensity of AEs was graded as mild, moderate, or severe. Serious AEs (SAEs), medically attended AEs, and AEs that led to withdrawal were recorded throughout the study (days 1–181). The investigators assessed the relationship of all unsolicited AEs and SAEs to vaccination.

### Immunogenicity Assessment

Blood samples were drawn prevaccination (day 1) and 30 and 60 days postvaccination (days 31 and 61; Figure 1). Sera were stored frozen (less than or equal to −18 °C) until analysis. Anti-GBS CPS immunoglobulin G antibodies for serotypes Ia, Ib, and III were measured at GSK using a multiplex immunoassay, instead of the per protocol planned enzyme-linked immunosorbent assay (ELISA) [20] because of unavailability of the ELISA. The multiplex immunoassay has been described e multiplex immunoassay will be described [26] and is summarized in the Supplementary Materials. The assay’s lower limits of quantitation (LLQs) were 0.233 µg/mL (Ia), 0.155 µg/mL (Ib), and 0.293 µg/mL (III). Serum samples for the baseline prevaccination time point of the parent study, previously analyzed by ELISA [22], were retested with the multiplex immunoassay to allow comparison with the concentrations obtained for the samples from the extension study.

### Statistical Analyses

The sample size was determined by the number of women from the parent study who agreed to participate in the extension study, plus 20 women who did not participate in the parent study.

The safety analysis was performed on the safety set, that is, all women vaccinated in the extension study and who provided postvaccination safety data. The primary safety endpoint was to assess the percentages of women who reported AEs over the time periods described above.

The immunogenicity analysis was performed on the per protocol immunogenicity set (PPS), that is, all women who had correctly received the study vaccine in the parent study (prior GBS and prior placebo groups) and received the study vaccine dose, complied with protocol-defined procedures, and had immunogenicity data available for at least 1 time point in the extension study. The primary immunogenicity endpoint was to assess the percentage of women who reached a range of arbitrary, prespecified serotype-specific anti-GBS concentration thresholds (0.5–8 µg/mL) 60 days postvaccination, reflecting the average time between third-trimester maternal vaccination and delivery. Secondary immunogenicity endpoints included the evaluation of percentages of women who reached these thresholds 30 days post-GBS vaccination and 30/60 days post-GBS vaccination by baseline serotype-specific anti-GBS concentrations (<LLQ). Baseline referred to the prevaccination time point in the parent study for the prior GBS and prior placebo groups and to the prevaccination time point (day 1) in the extension study for the naïve group (Figure 1). We computed percentages and 2-sided 95% Clopper-Pearson confidence intervals (CIs) for each group and different pooled groups (nonadjuvanted plus alum-adjuvanted, half- plus full-dosage MF59-adjuvanted, any prior GBS, and prior placebo plus naïve; Figure 1). Differences in percentages between the pooled prior GBS and pooled no prior GBS groups were calculated with 2-sided 95% CIs using the Miettinen and Nurminen method. Reverse cumulative distribution curves of anti-GBS concentrations were generated.

We also assessed serotype-specific geometric mean concentrations (GMCs) and within-subject geometric mean ratios (GMRs) 30 and 60 days postvaccination in all women and by baseline serotype-specific anti-GBS concentration (<LLQ or ≥LLQ). GMRs were determined relative to parent study baseline levels and relative to day 1 levels in the extension study. For GMC calculations, antibody concentrations <LLQ were given an arbitrary value of half the LLQ. Adjusted GMCs with 2-sided 95% CIs were calculated from log_10_-transformed antibody concentrations with an analysis of covariance model with vaccine group as the qualitative factor and log_10_-transformed baseline concentration as the covariate. Baseline and day 1 GMCs were calculated using an analysis of variance (ANOVA) model. GMRs with 2-sided 95% CIs were calculated from the log_10_-transformed within-subject ratios of antibody concentrations (postvaccination/prevaccination) using an ANOVA model with vaccine group as the qualitative factor.

The protocol included an assessment of antidiphtheria antibody levels to evaluate if the CRM_197_ carrier–lowered preexisting antidiphtheria titers. This analysis was canceled because no such interference was observed for other CRM_197_-conjugated vaccines [27, 28].

## RESULTS

### Study Participants

**Figure 2.**
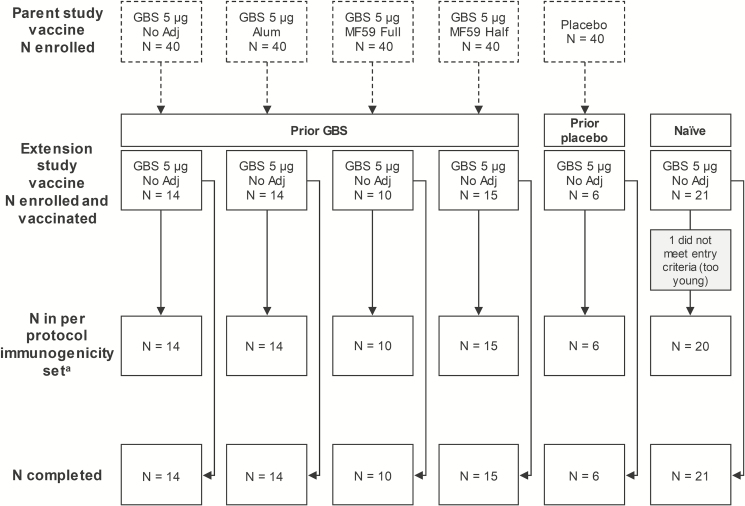
Participant flow diagram. Abbreviations: alum, aluminum hydroxide-adjuvanted formulation; GBS, group B streptococcus; MF59 full, full-dosage MF59-adjuvanted formulation; MF59 half, half-dosage MF59-adjuvanted formulation; no adj, nonadjuvanted formulation. ^a^One woman in the prior group B streptococcus (GBS) no adj group was excluded from the day 31 and day 61 immunogenicity analyses because she received a vaccine forbidden by the protocol on day 21 and no blood was drawn on day 61. One woman in the prior placebo group was excluded from the day 61 immunogenicity analysis because of noncompliance with the blood draw schedule. One woman in the prior GBS alum group and 1 in the prior GBS MF59 full-dosage group were excluded from the day 31 and day 61 immunogenicity analyses because the sample concentrations for serotypes III and Ia, respectively, could not be calculated due to nontitrable mean fluorescence intensity signals. Two women in the prior placebo group had no parent study baseline sample available.

A total of 80 women were enrolled in the extension study: 53 who had received 1 dose of the trivalent GBS vaccine 4–6 years earlier in the parent study (prior GBS groups) and 27 not previously GBS-vaccinated (no prior GBS groups), 6 of whom had received placebo in the parent study (prior placebo group) and 21 newly enrolled (naïve group). All enrolled women received 1 dose of trivalent GBS vaccine and completed the study (Figure 2). The PPS comprised 79 participants (Figure 2). Baseline characteristics and time between parent and extension study vaccinations were similar across groups (Table 1).

**Table 1.** Demographic Characteristics at Enrollment: All Enrolled Set

	Prior GBS				Prior Placebo	Naïve
Parent Study Vaccine	GBS No Adj	GBS Alum	GBS MF59 Full	GBS MF59 Half	Placebo	Not Applicable
Extension Study Vaccine	GBS No Adj	GBS No Adj	GBS No Adj	GBS No Adj	GBS No Adj	GBS No Adj
	N = 14	N = 14	N = 10	N = 15	N = 6	N = 21
Mean age ± SD, y	32.4 ± 6.9	31.6 ± 6.1	29.7 ± 6.2	28.9 ± 4.7	28.3 ± 5.4	29.2 ± 7.0
Caucasian ethnicity, n	14	14	10	15	6	21
Mean weight ± SD, kg	73.1 ± 12.5	63.4 ± 6.9	67.6 ± 12.3	62.9 ± 8.2	62.5 ± 7.9	62.8 ± 6.4
Mean height ± SD, cm	168.9 ± 5.4	166.1 ± 4.9	170.5 ± 5.1	164.4 ± 7.1	163.3 ± 5.0	169.0 ± 6.0
Mean body mass index ± SD, kg/m^2^	25.7 ± 4.6	23.0 ± 2.6	23.2 ± 3.4	23.3 ± 2.9	23.5 ± 4.0	22.0 ± 2.2
Mean time between parent and extension study vaccination ± SD, y	5.8 ± 0.06	5.8 ± 0.05	5.5 ± 0.05	5.5 ± 0.06	5.7 ± 0.13	NA

Abbreviations: alum, aluminum hydroxide-adjuvanted formulation; GBS, group B streptococcus; MF59 full, full-dosage MF59-adjuvanted formulation; MF59 half, half-dosage MF59-adjuvanted formulation; N, number of enrolled women in each group; n, number of women in the specified category; no adj, nonadjuvanted formulation; SD, standard deviation.

### Reactogenicity and Safety

Injection site pain was the most common solicited local AE (>50% of women across groups). The most frequent systemic AEs were fatigue and headache (Table 2). Most solicited AEs were mild or moderate. No fever was reported. Across groups, 29%–67% of women reported unsolicited AEs within 31 days postvaccination. Four women experienced unsolicited AEs considered as at least possibly related to vaccination: 2 in the prior GBS groups (upper respiratory tract infection, hot flush) and 2 in the no prior GBS groups (injection site erythema, nasal congestion; Table 2). These AEs were mild and resolved without sequelae. Throughout the study and across groups, 0%–47% of women reported unsolicited AEs that needed medical attention; none were deemed related to vaccination (Table 2). No SAEs were reported during the study, and no women withdrew due to AEs.

**Table 2.** Number (%) of Participants Reporting Adverse Events Postvaccination in the Extension Study: Safety Set

	Prior GBS				Prior Placebo	Naïve
Parent Study Vaccine	GBS No Adj	GBS Alum	GBS MF59 Full	GBS MF59 Half	Placebo	Not Applicable
Extension Study Vaccine	GBS No Adj	GBS No Adj	GBS No Adj	GBS No Adj	GBS No Adj	GBS No Adj
*Solicited AEs (days 1–7)*	N = 14	N = 14	N = 10	N = 15	N = 5	N = 21
Local						
Pain	10 (71%)	9 (64%)	6 (60%)	9 (60%)	3 (60%)	11 (52%)
Severe	1 (7%)	0	0	0	0	0
Erythema	0	0	0	0	1 (20%)	0
Swelling	0	1 (7%)	0	1 (7%)	1 (20%)	0
Warmth	1 (7%)	4 (29%)	2 (20%)	4 (27%)	2 (40%)	2 (10%)
Induration	0	0	0	1 (7%)	1 (20%)	0
Ecchymosis	0	0	0	0	0	0
Systemic						
Chills	0	0	1 (10%)	1 (7%)	1 (20%)	0
Nausea	0	2 (14%)	0	2 (13%)	0	1 (5%)
Severe	0	1 (7%)	0	0	0	0
Malaise	0	2 (14%)	2 (20%)	2 (13%)	1 (20%)	1 (5%)
Generalized myalgia	2 (14%)	2 (14%)	1 (10%)	2 (13%)	1 (20%)	1 (5%)
Severe	1 (7%)	0	0	0	0	0
Generalized arthralgia	0	1 (7%)	0	2 (13%)	0	0
Headache	4 (29%)	4 (29%)	2 (20%)	5 (33%)	2 (40%)	4 (19%)
Severe	0	2 (14%)	0	0	1 (20%)	1 (5%)
Fatigue	3 (21%)	10 (71%)	4 (40%)	4 (27%)	1 (20%)	2 (10%)
Body rash	0	1 (7%)	0	0	0	0
Fever (≥38°C)	0	0	0	0	0	0
*Unsolicited AEs (days 1–31)*	N = 14	N = 14	N = 10	N = 15	N = 6	N = 21
Any	4 (29%)	5 (36%)	4 (40%)	4 (27%)	4 (67%)	9 (43%)
At least possibly related^a^	1 (7%)	1 (7%)	0	0	1 (17%)	1 (5%)
*Unsolicited AEs (days 1–181)*	N = 14	N = 14	N = 10	N = 15	N = 6	N = 21
Serious	0	0	0	0	0	0
Medically attended	5 (36%)	5 (36%)	0	7 (47%)	2 (33%)	5 (24%)
** **Leading to withdrawal	0	0	0	0	0	0

A severe AE was defined as an AE that prevented normal daily activities or, for body rash, a rash that covered most of the skin. When no severe category is included for an AE in this table, no severe intensity was reported for that AE.

Abbreviations: AE, adverse event; alum, aluminum hydroxide-adjuvanted formulation; GBS, group B streptococcus; MF59 full, full-dosage MF59-adjuvanted formulation; MF59 half, half-dosage MF59-adjuvanted formulation; N, number of enrolled women in each group; no adj, nonadjuvanted formulation; SD, standard deviation.

^a^Includes possibly related and probably related AEs.

### Immunogenicity

**Figure 3.**
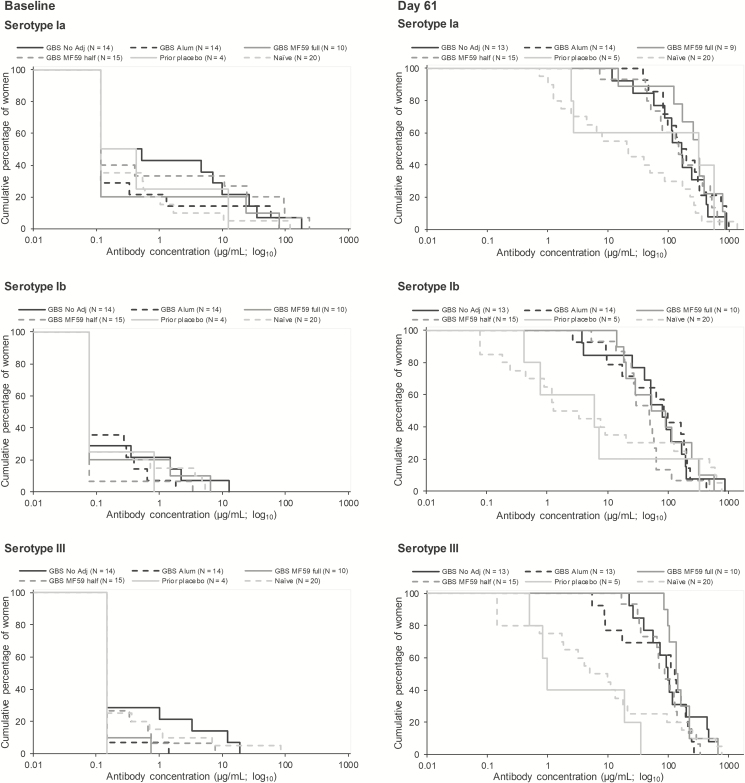
Reverse cumulative distribution curves of serotype-specific anti-GBS antibody concentrations at baseline and 60 days postvaccination (per protocol immunogenicity set). Analyses on all women regardless of their baseline lower limit of quantitation status. Baseline refers to the prevaccination time point in the parent study for the prior GBS and prior placebo groups and to the prevaccination time point (day 1) in the extension study for the naïve group. Abbreviations: alum, aluminum hydroxide-adjuvanted formulation; GBS, group B streptococcus; MF59 full, full-dosage MF59-adjuvanted formulation; MF59 half, half-dosage MF59-adjuvanted formulation; N, number of women with available results in each group; no adj, nonadjuvanted formulation.

All women in the PPS who had been vaccinated with trivalent GBS vaccine 4–6 years earlier had serotype-specific anti-GBS concentrations that exceeded an arbitrary threshold of 1 µg/mL for serotypes Ia, Ib, and III 60 days after receiving their second GBS vaccine dose in the extension study (Table 3, Supplementary Table 1). In addition, 92%–98% of women in the pooled prior GBS group reached an arbitrary threshold of 8 µg/mL 60 days postdose 2 across serotypes. This was substantially higher than percentages that achieved that threshold 60 days postdose 1 among previously non–GBS-vaccinated women (36%–56%; Table 3). Similar trends were noted across the other thresholds and for the 30 days postvaccination time point, with higher percentages reaching these thresholds after a second rather than after a first dose (Supplementary Table 1). The GBS vaccine formulation used in the parent study did not appear to have an observable impact on these percentages (Supplementary Table 1). Reverse cumulative distribution curves confirm these results (Figure 3, Supplementary Figure 1).

**Table 3.** Percentages of Women With Serotype-specific Anti-group B Streptococcus Antibody Concentrations ≥1 µg/mL and ≥8 µg/mL in the Pooled Groups and Group Differences 60 Days Postvaccination, per Protocol Immunogenicity Set

	Percentage of Women ≥ Threshold (95% CI)				
	Prior GBS	Prior GBS	Prior GBS		
	No Adj/Alum	MF59	Any	No Prior GBS	Group Difference (Prior GBS Any–No Prior GBS) % (95% CI)
*GBS serotype Ia*					
All	N = 27	N = 24	N = 51	N = 25	
% ≥1 µg/mL	100 (87.2, 100)	100 (85.8, 100)	100 (93.0, 100)	92 (74.0, 99.0)	8 (0.5, 25.1)
% ≥8 µg/mL	100 (87.2, 100)	96 (78.9, 99.9)	98 (89.6, 100)	56 (34.9, 75.6)	42 (23.8, 61.4)
<LLQ	N = 17	N = 16	N = 33	N = 15	
% ≥1 µg/mL	100 (80.5, 100)	100 (79.4, 100)	100 (89.4, 100)	93 (68.1, 99.8)	7 (−4.5, 30.1)
% ≥8 µg/mL	100 (80.5, 100)	94 (69.8, 99.8)	97 (84.2, 99.9)	47 (21.3, 73.4)	50 (25.4, 73.0)
*GBS serotype Ib*					
All	N = 27	N = 25	N = 52	N = 25	
% ≥1 µg/mL	100 (87.2, 100)	100 (86.3, 100)	100 (93.2, 100)	64 (42.5, 82.0)	36 (20.2, 55.6)
% ≥8 µg/mL	89 (70.8, 97.6)	96 (79.6, 99.9)	92 (81.5, 97.9)	36 (18.0, 57.5)	56 (34.8, 73.5)
<LLQ	N = 19	N = 22	N = 41	N = 18	
% ≥1 µg/mL	100 (82.4, 100)	100 (84.6, 100)	100 (91.4, 100)	50 (26.0, 74.0)	50 (28.9, 71.1)
% ≥8 µg/mL	84 (60.4, 96.6)	95 (77.2, 99.9)	90 (76.9, 97.3)	17 (3.6, 41.4)	74 (48.6, 87.3)
*GBS serotype III*					
All	N = 26	N = 25	N = 51	N = 25	
% ≥1 µg/mL	100 (86.8, 100)	100 (86.3, 100)	100 (93.0, 100)	68 (46.5, 85.1)	32 (17.1, 51.7)
% ≥8 µg/mL	96 (80.4, 99.9)	100 (86.3, 100)	98 (89.6, 100)	48 (27.8, 68.7)	50 (30.7, 68.4)
<LLQ	N = 22	N = 20	N = 42	N = 19	
% ≥1 µg/mL	100 (84.6, 100)	100 (83.2, 100)	100 (91.6, 100)	58 (33.5, 79.7)	42 (23.0, 63.9)
% ≥8 µg/mL	95 (77.2, 99.9)	100 (83.2, 100)	98 (87.4, 99.9)	37 (16.3, 61.6)	61 (37.7, 79.1)

Abbreviations: CI, confidence interval; GBS, group B streptococcus; LLQ, lower limit of quantitation; N, number of women with available results in each group.

See Figure 1 for group names. Analyses on all women regardless of their baseline LLQ status (“all”) and on women with baseline serotype-specific anti-GBS antibody concentrations below the LLQs (“<LLQ”). Baseline refers to the prevaccination time point in the parent study for the prior GBS and prior placebo groups and to the prevaccination time point in the current extension study (day 1) for the naïve group.

**Figure 4.**
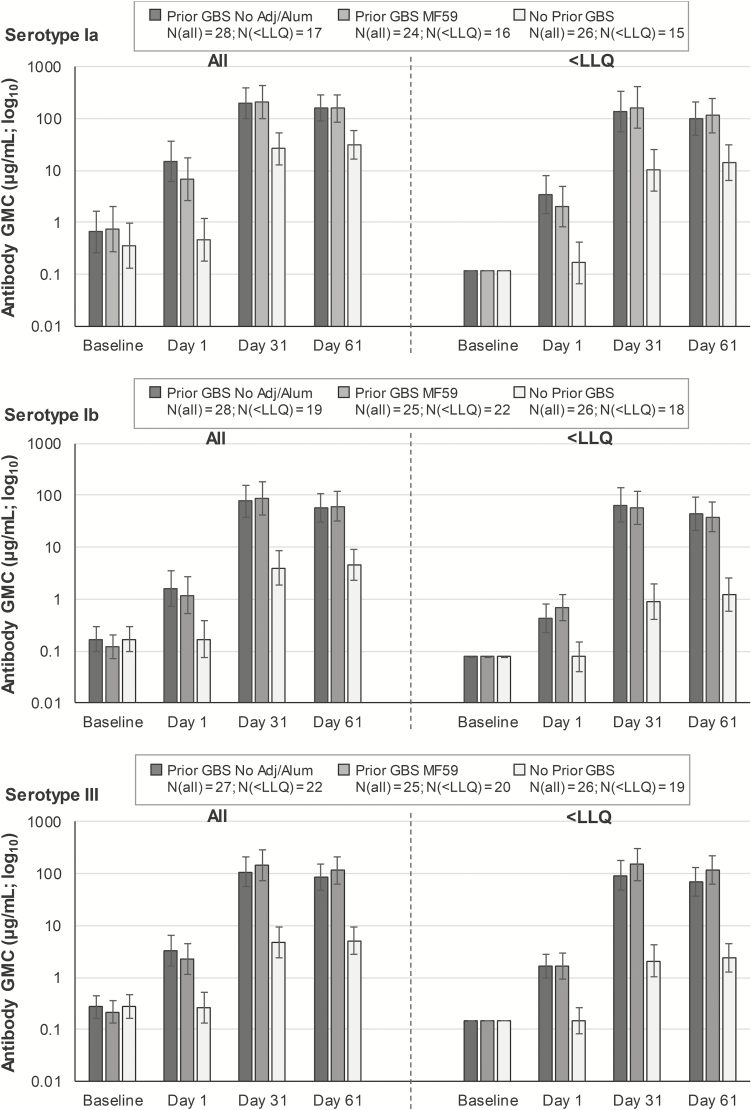
Serotype-specific geometric mean anti-GBS antibody concentrations at different time points for the pooled groups (per protocol immunogenicity set). See Figure 1 for group names. Analyses on all women regardless of their baseline lower limit of quantitation (LLQ) status (“all,” left side) and on women with baseline serotype-specific anti-GBS antibody concentrations below the LLQs (“<LLQ,” right side). Baseline refers to the prevaccination time point in the parent study for the prior GBS and prior placebo groups and to the prevaccination time point in the extension study (day 1) for the naïve group. Antibody concentrations <LLQ were given an arbitrary value of half the LLQ. Day 1 refers to the prevaccination time point in the extension study for all groups. Confidence intervals are depicted as error bars. Abbreviations: GBS, group B streptococcus; GMC, geometric mean concentration; LLQ, lower limit of quantitation; N, maximum number of women with available results across time points.

For each serotype, anti-GBS GMCs 30/60 days postdose 2 (prior GBS groups) were substantially higher than baseline GMCs (approximately 200- to 600-fold) and GMCs 30/60 days postdose 1 in the no prior GBS group (approximately 5- to 30-fold; Figure 4, Supplementary Table 2). No significant differences in postdose 2 antibody GMCs were observed between the prior GBS groups based on the vaccine formulation received as the first dose in the parent study (Supplementary Table 2). Significant increases (approximately 20- to 70-fold) in antibody GMCs compared to baseline were also observed for each serotype 30/60 days postdose 1 in the no prior GBS groups (Figure 4, Supplementary Table 2).

In previous studies, women with undetectable prevaccination concentrations mounted a less robust response to GBS vaccination [20–23, 29]. We therefore assessed the anti-GBS response based on whether women had detectable or undetectable antibody concentrations at baseline (ie, above or below the serotype-specific LLQs before the first GBS vaccine dose). Most women across groups included in our analysis had baseline antibody concentrations <LLQs (49/77 [64%] for Ia, 59/77 [77%] for Ib, and 62/77 [81%] for III). Also, 90%–98% of women with undetectable baseline concentrations in the pooled prior GBS group reached the 8-µg/mL threshold 60 days postdose 2 across serotypes (Table 3), with similar percentages for the other thresholds and 30 days postdose 2 (Supplementary Table 1). Previously GBS-vaccinated women with undetectable baseline antibody concentrations showed higher antibody GMCs 30/60 days postdose 2 vs baseline and vs GMCs 30/60 days postdose 1 in previously non–GBS-vaccinated women (approximately 7- to 70-fold; Figure 4, Supplementary Table 3). Among the small subset of women with detectable baseline antibody concentrations, serotype-specific anti-GBS GMCs were high 30/60 days postdose 1 in the no prior GBS groups and postdose 2 in the prior GBS groups, without consistent differences between postdose 1 and postdose 2 responses (Supplementary Table 3).

## DISCUSSION

We are the first to evaluate the safety and immunogenicity of a second dose of the investigational trivalent GBS vaccine given at an interval after the first dose close to the average interpregnancy interval in some populations [30, 31]. Our study did not reveal any tolerability or safety concerns of a second dose given 4–6 years postfirst dose. The second dose elicited a robust immune response, particularly in women with undetectable antibody levels prefirst dose. In these women, a second vaccine dose induced more robust responses than a first dose.

While an inverse relation between maternal serotype-specific anti-GBS levels and the risk of invasive GBS disease in young infants has been demonstrated [6, 14–19], there is no established serological correlate of protection against invasive GBS disease in young infants. The absence of a standardized anti-GBS immunoassay complicates the establishment of a correlate of protection [11, 17, 32]. We therefore assessed several arbitrary thresholds between 0.5 and 8 µg/mL (close to the range of proposed seroprotection thresholds for other assays [1–10 µg/mL] [11, 14, 15, 17–19, 32–34]). Nearly all women reached the 8-µg/mL threshold after a second dose regardless of their baseline antibody levels, while a notably smaller proportion (≤50% 60 days postvaccination) reached this threshold after a single dose among women with undetectable baseline antibody levels.

Few studies have assessed the response to 2 GBS vaccine doses [11, 22, 23, 29]. The parent study compared groups receiving 1 or 2 doses (administered 1 month apart) of various trivalent GBS vaccine formulations and found no clear differences across groups in serotype-specific antibody GMCs postvaccination [22]. A second dose did not improve the immune response in women with undetectable baseline antibody levels, although subgroup sizes were small, making it difficult to draw definite conclusions [22]. A study in South Africa compared immune responses 1 month postfirst and postsecond dose in women receiving a 2-dose trivalent GBS vaccine regimen with doses spaced 1 month apart. No increases in antibody GMCs were observed after a second dose. No analysis by baseline LLQ status was performed [23]. A beneficial effect of a second dose given 21 months after first vaccination was demonstrated in a phase 1 study with a monovalent GBS serotype III CPS tetanus toxoid conjugate vaccine (GBS III-TT) [29]. While postdose 2 serotype III-specific antibody GMCs were similar to postdose 1 GMCs in the overall study population, in the small subset of participants (n = 8) with undetectable prevaccination antibody concentrations, the serotype III-specific GMC postdose 2 was 3-fold higher than postdose 1 [29]. This is consistent with what was observed in our study, where the subset of participants with undetectable baseline antibody levels was greater (64%–81% vs 22% in the GBS III-TT study). These results indicate that women with undetectable prevaccination anti-GBS concentrations, previously shown to mount a less robust response to GBS vaccination [20–23, 29], may benefit from receiving a sufficiently spaced second GBS vaccine dose.

In the parent study, no additional benefit from adding alum or MF59 adjuvant could be shown [22]. Likewise, we observed no major effect of the vaccine formulation used in the parent study on the immune response after a second dose given 4–6 years later. The small sample size warrants caution when interpreting these results.

As GBS vaccination is intended for use during pregnancy, a favorable reactogenicity and safety profile is essential [13]. In our study, severe solicited AEs were rare and no fever or SAEs were reported. Four women experienced mild, nonserious unsolicited AEs considered as possibly or probably vaccination-related. While the number of participants per group was too small for conclusive group comparisons, no obvious differences were observed between the rates of solicited or unsolicited AEs after a second or first trivalent GBS vaccine dose. Likewise, reactogenicity after a second dose in the extension study was similar to that of 1 or 2 doses of the 5-µg nonadjuvanted formulation in the parent study [22].

Aside from the small sample size and descriptive nature of the analyses, our study has other limitations. We only assessed a 4- to 6-year interval between doses, which is longer than the interpregnancy interval in many settings [29, 30, 34], and we did not establish the minimum interval required for the second vaccine dose to mount a robust immune response. No information was collected on whether women became GBS-colonized/exposed or were pregnant between the parent and extension studies. All participants were white; results may therefore not be generalizable across populations or ethnicities. As we used a new assay and different standard sera for quantification of serum antibodies, results are hard to compare with related studies that used the trivalent vaccine [20–24]. In the absence of a correlate of protection, we cannot conclude on the effect of a second dose on protection against infant invasive GBS disease. However, given the proven association between maternal serotype-specific anti-GBS levels and protection [6, 14–19], a second GBS vaccine dose given at a 4- to 6-year interval is likely to improve protection among infants whose mothers were seronegative before initial vaccination.

In summary, our results show that a second dose of the investigational trivalent GBS vaccine administered 4–6 years after a first dose had a favorable safety profile and was immunogenic, regardless of baseline antibody concentrations. A 2-dose schedule with doses spaced far enough apart may be beneficial for women with very low preexisting antibody concentrations.

## Supplementary Data

Supplementary materials are available at *Clinical Infectious Diseases* online. Consisting of data provided by the authors to benefit the reader, the posted materials are not copyedited and are the sole responsibility of the authors, so questions or comments should be addressed to the corresponding author.

ciz737_suppl_Supplementary_Material

## Notes

***Author contributions.*** G. L. R., Z. B., I. M., A. K., S. C., and K. S. designed the study. G. L. R., Z. B., C. M., A. A., F. D. B., G. B., I. M., and A. K. acquired the data. G. L. R., Z. B., L. G., G. B., I. M., A. K., B. C., and O. H. analyzed the data. G. L. R., C. M., A. A., F. D. B., A. K., and B. C. contributed to the conduct of the study. All authors reviewed and revised the manuscript and approved the final manuscript as submitted.

***Acknowledgments.*** The authors are grateful to the women who participated in the study. We thank the research program assistants and study nurses for their dedicated efforts on this trial and the clinical and serological laboratory teams of the GSK group of companies for their contribution to this study. The authors also thank Natalie Denef (Modis c/o GSK) for medical writing support and Stéphanie Deroo (Modis c/o GSK) for manuscript coordination. MF59 is a trademark of Novartis. The protocol and a results summary for this study (GSK study 205421–NCT02690181) are available on the GSK Clinical Study Register and can be accessed at https://www.gsk-studyregister.com/. For interventional studies that evaluate GSK medicines, anonymized patient-level data will be made available to independent researchers, subject to review by an independent panel, at www.clinicalstudydatarequest.com within 6 months of publication. To protect the privacy of patients and individuals involved in our studies, GSK does not publicly disclose patient-level data.

***Financial support.*** This work was supported by Novartis Vaccines Division and GlaxoSmithKline Biologicals SA. On 2 March 2015, Novartis’ noninfluenza vaccines business was acquired by the GSK group of companies. GlaxoSmithKline Biologicals SA paid for all costs associated with the development and publishing of this manuscript.

***Potential conflicts of interest.*** Z. B., L. G., G. B., I. M., A. K., B. C., and O. H. are employees of the GSK group of companies. I. M., A. K., B. C., and O. H. hold shares in the GSK group of companies. K. S. was an employee of Novartis Vaccines Division and the GSK group of companies. S. C. was an employee of Novartis Vaccines Division and the GSK group of companies and is currently an employee of Novartis and holds shares in the GSK group of companies and Novartis. I. M. is listed as inventor on patents owned by the GSK group of companies. G. L. R. and A. A. report that their institutions have been financially compensated for the conduct of the study. The other authors report no potential conflicts. All authors have submitted the ICMJE Form for Disclosure of Potential Conflicts of Interest. Conflicts that the editors consider relevant to the content of the manuscript have been disclosed.
