# Supplementary material for: Safety and Immunogenicity of a Second Dose of an Investigational Maternal Trivalent Group B Streptococcus Vaccine in Nonpregnant Women 4–6 Years After a First Dose: Results From a Phase 2 Trial
Source: Clin Infect Dis. 2019 Aug 8;70(12):2570–9. doi: 10.1093/cid/ciz737 (PMC7286364; doi:10.1093/cid/ciz737)
Supplement: ciz737_suppl_Supplementary_Material [file ciz737_suppl_supplementary_material.docx]

**Supplementary Material**

**Supplementary Methods**

**Inclusion criteria**

1. Healthy, non-pregnant women who had received a single 5 μg dose of trivalent Group B streptococcus (GBS) vaccine or placebo in the parent study and healthy non-pregnant women aged 22–46 years inclusive on the day of informed consent who had not received any GBS vaccine in the past.
2. Individuals who voluntarily gave written informed consent after the nature of the study had been explained according to local regulatory requirements, prior to study entry.
3. Individuals in good health as determined by the outcome of medical history, physical examination and clinical judgment of the investigator.
4. Individuals who could comply with study procedures including follow-up.
5. Women of childbearing potential who were using an effective birth control method which they intended to use until study end (day 181 visit) or women of nonchildbearing potential.

**Exclusion criteria**

1. Progressive, unstable or uncontrolled clinical conditions.
2. Clinical conditions representing a contraindication to intramuscular vaccination and blood draws.
3. Abnormal function of the immune system resulting from:
   1. Clinical conditions, including but not limited to known or suspected HIV infection or HIV-related disease, a history of or an active autoimmune disorder (as judged by the investigator)
   2. Systemic administration of corticosteroids (per os/intravenously/intramuscularly) for more than 14 consecutive days within 90 days prior to informed consent.
   3. Administration of antineoplastic and immunomodulating agents or radiotherapy within 90 days prior to informed consent.
   4. Receipt of immunosuppressive therapy within 90 days prior to informed consent
4. Received immunoglobulins or any blood products within 180 days prior to informed consent.
5. Received an investigational or non-registered medicinal product within 30 days prior to informed consent.
6. Immediate family or household member of study personnel.
7. Any other clinical condition that, in the opinion of the investigator, might interfere with the results of the study or pose additional risk to the individual due to participation in the study.
8. Individuals who received any other vaccines within 14 days for inactivated vaccines or 28 days for live vaccines prior to enrolment in this study or who were planning to receive any vaccine within 28 days from the study vaccination. Exception: an inactivated influenza vaccine could be administered up to 7 days prior to study vaccination or 7 days after study vaccination.
9. Individuals who anticipated becoming pregnant prior to study end (day 181 visit).
10. Individuals who were nursing (breastfeeding).
11. Individuals who had a previous immunization with a vaccine containing GBS antigens that was not part of the parent study.
12. Individuals with a fever (oral temperature ≥38°C) within 3 days prior to day 1 or use of antipyretics and/or analgesic medications within 24 hours prior to day 1.
13. Individuals with acute or chronic infection(s) that required systemic antibiotic treatment or antiviral therapy, within 7 days prior to day 1.
14. Individuals with a history of severe allergic reactions after previous vaccinations or medications, such as anaphylactic shock, asthma, urticaria, or other allergic reaction or hypersensitivity to any vaccine component or medical equipment including latex used in this study.
15. Individuals with any progressive or severe neurologic disorder, seizure disorder, epilepsy or Guillain-Barré syndrome.
16. Individuals with history of substance or alcohol abuse within the past 2 years.

**Multiplex immunoassay**

The multiplex immunoassay is based on the *Luminex* technology and uses biotin-derivatized capsular polysaccharide (CPS) of five GBS serotypes (Ia, Ib, II, III and V) each coupled to streptavidin magnetic beads containing a different fluorescent dye. The assay development will be described elsewhere (manuscript in preparation). Eleven serial serum dilutions were added to the coupled bead mixture in 96-well microplates (Greiner, Millipore Corp.) and incubated for 90 minutes at room temperature. Beads were washed twice with phosphate buffered saline (PBS) and incubated with 2.5 µg/mL of R-phycoerythrin-conjugated goat anti-human immunoglobulin G (IgG) secondary antibody (Jackson Immunoresearch) for 60 minutes. Beads were washed twice and resuspended in PBS. Fluorescence analysis was performed on a FlexMAP3D instrument by using BioplexManager software version 6.1 (Biorad) and mean fluorescent intensities were converted to IgG concentrations in µg/mL by interpolation from a 5-parameter logistic standard curve for every bead/serotype. A sub-standard serum pool was prepared from GBS-infected adult sera [1] and calibrated against monovalent weighted sera [2, 3].

1. Edwards MS, Rench MA, Rinaudo CD, et al. Immune responses to invasive group B streptococcal disease in adults. *Emerg Infect Dis* 2016; 22(11):1877–83.
2. Baker CJ, Carey VJ, Rench MA, et al. Maternal antibody at delivery protects neonates from early onset group B streptococcal disease. *J Infect Dis* 2014; 209(5):781–8.
3. Guttormsen HK, Baker CJ, Edwards MS, Paoletti LC, Kasper DL. Quantitative determination of antibodies to type III group B streptococcal polysaccharide. *J Infect Dis* 1996; 173(1):142–50.

**Supplementary Tables**

**Supplementary Table 1. Percentages of women (95% confidence intervals) with serotype-specific anti-GBS antibody concentrations above different thresholds pre-vaccination and 30 and 60 days post-vaccination in each group (per protocol immunogenicity set)**

|  |  | **Prior GBS** |  |  |  | **Prior placebo** |  | **Naïve** |
| --- | --- | --- | --- | --- | --- | --- | --- | --- |
| **Parent study vaccine:** | **GBS No Adj** | **GBS Alum** | **GBS MF59 Full** | **GBS MF59 Half** |  | **Placebo** |  | **NA** |
| **Extension study vaccine:** | **GBS No Adj** | **GBS No Adj** | **GBS No Adj** | **GBS No Adj** |  | **GBS No Adj** |  | **GBS No Adj** |
| ***Serotype Ia*** |  |  |  |  |  |  |  |  |
| **All** |  |  |  |  |  |  |  |  |
| **Day 1 (pre-vaccination)** | N=14 | N=14 | N=10 | N=15 |  | N=6 |  | N=20 |
| % ≥0.5 µg/mL | 86 (57.2, 98.2) | 93 (66.1, 99.8) | 70 (34.8, 93.3) | 87 (59.5, 98.3) |  | 50 (11.8, 88.2) |  | 35 (15.4, 59.2) |
| % ≥1 µg/mL | 86 (57.2, 98.2) | 86 (57.2, 98.2) | 70 (34.8, 93.3) | 73 (44.9, 92.2) |  | 50 (11.8, 88.2) |  | 20 (5.7, 43.7) |
| % ≥2 µg/mL | 86 (57.2, 98.2) | 79 (49.2, 95.3) | 50 (18.7, 81.3) | 73 (44.9, 92.2) |  | 33 (4.3, 77.7) |  | 10 (1.2, 31.7) |
| % ≥3 µg/mL | 79 (49.2, 95.3) | 71 (41.9, 91.6) | 50 (18.7, 81.3) | 60 (32.3, 83.7) |  | 33 (4.3, 77.7) |  | 10 (1.2, 31.7) |
| % ≥5 µg/mL | 71 (41.9, 91.6) | 57 (28.9, 82.3) | 50 (18.7, 81.3) | 40 (16.3, 67.7) |  | 33 (4.3, 77.7) |  | 10 (1.2, 31.7) |
| % ≥8 µg/mL | 64 (35.1, 87.2) | 57 (28.9, 82.3) | 50 (18.7, 81.3) | 40 (16.3, 67.7) |  | 33 (4.3, 77.7) |  | 10 (1.2, 31.7) |
| **30 days post-vaccination** | N=13 | N=14 | N=9 | N=15 |  | N=6 |  | N=20 |
| % ≥0.5 µg/mL | 100 (75.3, 100) | 100 (76.8, 100) | 100 (66.4, 100) | 100 (78.2, 100) |  | 100 (54.1, 100) |  | 85 (62.1, 96.8) |
| % ≥1 µg/mL | 100 (75.3, 100) | 100 (76.8, 100) | 100 (66.4, 100) | 100 (78.2, 100) |  | 100 (54.1, 100) |  | 70 (45.7, 88.1) |
| % ≥2 µg/mL | 100 (75.3, 100) | 100 (76.8, 100) | 100 (66.4, 100) | 100 (78.2, 100) |  | 67 (22.3, 95.7) |  | 65 (40.8, 84.6) |
| % ≥3 µg/mL | 100 (75.3, 100) | 100 (76.8, 100) | 100 (66.4, 100) | 100 (78.2, 100) |  | 67 (22.3, 95.7) |  | 60 (36.1, 80.9) |
| % ≥5 µg/mL | 100 (75.3, 100) | 100 (76.8, 100) | 100 (66.4, 100) | 100 (78.2, 100) |  | 67 (22.3, 95.7) |  | 60 (36.1, 80.9) |
| % ≥8 µg/mL | 100 (75.3, 100) | 100 (76.8, 100) | 100 (66.4, 100) | 100 (78.2, 100) |  | 67 (22.3, 95.7) |  | 60 (36.1, 80.9) |
| **60 days post-vaccination** | N=13 | N=14 | N=9 | N=15 |  | N=5 |  | N=20 |
| % ≥0.5 µg/mL | 100 (75.3, 100) | 100 (76.8, 100) | 100 (66.4, 100) | 100 (78.2, 100) |  | 100 (47.8, 100) |  | 100 (83.2, 100) |
| % ≥1 µg/mL | 100 (75.3, 100) | 100 (76.8, 100) | 100 (66.4, 100) | 100 (78.2, 100) |  | 100 (47.8, 100) |  | 90 (68.3, 98.8) |
| % ≥2 µg/mL | 100 (75.3, 100) | 100 (76.8, 100) | 100 (66.4, 100) | 100 (78.2, 100) |  | 100 (47.8, 100) |  | 75 (50.9, 91.3) |
| % ≥3 µg/mL | 100 (75.3, 100) | 100 (76.8, 100) | 100 (66.4, 100) | 100 (78.2, 100) |  | 60 (14.7, 94.7) |  | 70 (45.7, 88.1) |
| % ≥5 µg/mL | 100 (75.3, 100) | 100 (76.8, 100) | 100 (66.4, 100) | 100 (78.2, 100) |  | 60 (14.7, 94.7) |  | 65 (40.8, 84.6) |
| % ≥8 µg/mL | 100 (75.3, 100) | 100 (76.8, 100) | 100 (66.4, 100) | 93 (68.1, 99.8) |  | 60 (14.7, 94.7) |  | 55 (31.5, 76.9) |
| **<LLQ** |  |  |  |  |  |  |  |  |
| **Day 1 (pre-vaccination)** | N=7 | N=10 | N=8 | N=9 |  | N=2 |  | N=13 |
| % ≥0.5 µg/mL | 71 (29.0, 96.3) | 90 (55.5, 99.8) | 63 (24.5, 91.5) | 78 (40.0, 97.2) |  | 50 (1.3, 98.7) |  | 0 (0.0, 24.7) |
| % ≥1 µg/mL | 71 (29.0, 96.3) | 80 (44.4, 97.5) | 63 (24.5, 91.5) | 56 (21.2, 86.3) |  | 50 (1.3, 98.7) |  | 0 (0.0, 24.7) |
| % ≥2 µg/mL | 71 (29.0, 96.3) | 70 (34.8, 93.3) | 38 (8.5, 75.5) | 56 (21.2, 86.3) |  | 50 (1.3, 98.7) |  | 0 (0.0, 24.7) |
| % ≥3 µg/mL | 57 (18.4, 90.1) | 60 (26.2, 87.8) | 38 (8.5, 75.5) | 44 (13.7, 78.8) |  | 50 (1.3, 98.7) |  | 0 (0.0, 24.7) |
| % ≥5 µg/mL | 43 (9.9, 81.6) | 40 (12.2, 73.8) | 38 (8.5, 75.5) | 11 (0.3, 48.2) |  | 50 (1.3, 98.7) |  | 0 (0.0, 24.7) |
| % ≥8 µg/mL | 29 (3.7, 71.0) | 40 (12.2, 73.8) | 38 (8.5, 75.5) | 11 (0.3, 48.2) |  | 50 (1.3, 98.7) |  | 0 (0.0, 24.7) |
| **30 days post-vaccination** | N=7 | N=10 | N=7 | N=9 |  | N=2 |  | N=13 |
| % ≥0.5 µg/mL | 100 (59.0, 100) | 100 (69.2, 100) | 100 (59.0, 100) | 100 (66.4, 100) |  | 100 (15.8, 100) |  | 85 (54.6, 98.1) |
| % ≥1 µg/mL | 100 (59.0, 100) | 100 (69.2, 100) | 100 (59.0, 100) | 100 (66.4, 100) |  | 100 (15.8, 100) |  | 69 (38.6, 90.9) |
| % ≥2 µg/mL | 100 (59.0, 100) | 100 (69.2, 100) | 100 (59.0, 100) | 100 (66.4, 100) |  | 50 (1.3, 98.7) |  | 62 (31.6, 86.1) |
| % ≥3 µg/mL | 100 (59.0, 100) | 100 (69.2, 100) | 100 (59.0, 100) | 100 (66.4, 100) |  | 50 (1.3, 98.7) |  | 54 (25.1, 80.8) |
| % ≥5 µg/mL | 100 (59.0, 100) | 100 (69.2, 100) | 100 (59.0, 100) | 100 (66.4, 100) |  | 50 (1.3, 98.7) |  | 54 (25.1, 80.8) |
| % ≥8 µg/mL | 100 (59.0, 100) | 100 (69.2, 100) | 100 (59.0, 100) | 100 (66.4, 100) |  | 50 (1.3, 98.7) |  | 54 (25.1, 80.8) |
| **60 days post-vaccination** | N=7 | N=10 | N=7 | N=9 |  | N=2 |  | N=13 |
| % ≥0.5 µg/mL | 100 (59.0, 100) | 100 (69.2, 100) | 100 (59.0, 100) | 100 (66.4, 100) |  | 100 (15.8, 100) |  | 100 (75.3, 100) |
| % ≥1 µg/mL | 100 (59.0, 100) | 100 (69.2, 100) | 100 (59.0, 100) | 100 (66.4, 100) |  | 100 (15.8, 100) |  | 92 (64.0, 99.8) |
| % ≥2 µg/mL | 100 (59.0, 100) | 100 (69.2, 100) | 100 (59.0, 100) | 100 (66.4, 100) |  | 100 (15.8, 100) |  | 77 (46.2, 95.0) |
| % ≥3 µg/mL | 100 (59.0, 100) | 100 (69.2, 100) | 100 (59.0, 100) | 100 (66.4, 100) |  | 50 (1.3, 98.7) |  | 69 (38.6, 90.9) |
| % ≥5 µg/mL | 100 (59.0, 100) | 100 (69.2, 100) | 100 (59.0, 100) | 100 (66.4, 100) |  | 50 (1.3, 98.7) |  | 62 (31.6, 86.1) |
| % ≥8 µg/mL | 100 (59.0, 100) | 100 (69.2, 100) | 100 (59.0, 100) | 89 (51.8, 99.7) |  | 50 (1.3, 98.7) |  | 46 (19.2, 74.9) |
| ***Serotype Ib*** |  |  |  |  |  |  |  |  |
| **All** |  |  |  |  |  |  |  |  |
| **Day 1 (pre-vaccination)** | N=14 | N=14 | N=10 | N=15 |  | N=6 |  | N=20 |
| % ≥0.5 µg/mL | 57 (28.9, 82.3) | 64 (35.1, 87.2) | 70 (34.8, 93.3) | 67 (38.4, 88.2) |  | 17 (0.4, 64.1) |  | 20 (5.7, 43.7) |
| % ≥1 µg/mL | 50 (23.0, 77.0) | 57 (28.9, 82.3) | 50 (18.7, 81.3) | 40 (16.3, 67.7) |  | 17 (0.4, 64.1) |  | 15 (3.2, 37.9) |
| % ≥2 µg/mL | 29 (8.4, 58.1) | 50 (23.0, 77.0) | 40 (12.2, 73.8) | 27 (7.8, 55.1) |  | 17 (0.4, 64.1) |  | 15 (3.2, 37.9) |
| % ≥3 µg/mL | 29 (8.4, 58.1) | 50 (23.0, 77.0) | 40 (12.2, 73.8) | 27 (7.8, 55.1) |  | 17 (0.4, 64.1) |  | 15 (3.2, 37.9) |
| % ≥5 µg/mL | 29 (8.4, 58.1) | 50 (23.0, 77.0) | 30 (6.7, 65.2) | 20 (4.3, 48.1) |  | 17 (0.4, 64.1) |  | 5 (0.1, 24.9) |
| % ≥8 µg/mL | 29 (8.4, 58.1) | 50 (23.0, 77.0) | 30 (6.7, 65.2) | 7 (0.2, 31.9) |  | 0 (0.0, 45.9) |  | 0 (0.0, 16.8) |
| **30 days post-vaccination** | N=13 | N=14 | N=10 | N=15 |  | N=6 |  | N=20 |
| % ≥0.5 µg/mL | 100 (75.3, 100) | 100 (76.8, 100) | 100 (69.2, 100) | 100 (78.2, 100) |  | 83 (35.9, 99.6) |  | 60 (36.1, 80.9) |
| % ≥1 µg/mL | 100 (75.3, 100) | 100 (76.8, 100) | 100 (69.2, 100) | 100 (78.2, 100) |  | 83 (35.9, 99.6) |  | 45 (23.1, 68.5) |
| % ≥2 µg/mL | 100 (75.3, 100) | 100 (76.8, 100) | 100 (69.2, 100) | 100 (78.2, 100) |  | 50 (11.8, 88.2) |  | 45 (23.1, 68.5) |
| % ≥3 µg/mL | 100 (75.3, 100) | 93 (66.1, 99.8) | 100 (69.2, 100) | 100 (78.2, 100) |  | 50 (11.8, 88.2) |  | 45 (23.1, 68.5) |
| % ≥5 µg/mL | 92 (64.0, 99.8) | 93 (66.1, 99.8) | 100 (69.2, 100) | 100 (78.2, 100) |  | 50 (11.8, 88.2) |  | 45 (23.1, 68.5) |
| % ≥8 µg/mL | 85 (54.6, 98.1) | 93 (66.1, 99.8) | 100 (69.2, 100) | 93 (68.1, 99.8) |  | 50 (11.8, 88.2) |  | 45 (23.1, 68.5) |
| **60 days post-vaccination** | N=13 | N=14 | N=10 | N=15 |  | N=5 |  | N=20 |
| % ≥0.5 µg/mL | 100 (75.3, 100) | 100 (76.8, 100) | 100 (69.2, 100) | 100 (78.2, 100) |  | 80 (28.4, 99.5) |  | 70 (45.7, 88.1) |
| % ≥1 µg/mL | 100 (75.3, 100) | 100 (76.8, 100) | 100 (69.2, 100) | 100 (78.2, 100) |  | 60 (14.7, 94.7) |  | 65 (40.8, 84.6) |
| % ≥2 µg/mL | 100 (75.3, 100) | 100 (76.8, 100) | 100 (69.2, 100) | 100 (78.2, 100) |  | 60 (14.7, 94.7) |  | 50 (27.2, 72.8) |
| % ≥3 µg/mL | 100 (75.3, 100) | 93 (66.1, 99.8) | 100 (69.2, 100) | 100 (78.2, 100) |  | 60 (14.7, 94.7) |  | 50 (27.2, 72.8) |
| % ≥5 µg/mL | 85 (54.6, 98.1) | 93 (66.1, 99.8) | 100 (69.2, 100) | 100 (78.2, 100) |  | 60 (14.7, 94.7) |  | 45 (23.1, 68.5) |
| % ≥8 µg/mL | 85 (54.6, 98.1) | 93 (66.1, 99.8) | 100 (69.2, 100) | 93 (68.1, 99.8) |  | 20 (0.5, 71.6) |  | 40 (19.1, 63.9) |
| **<LLQ** |  |  |  |  |  |  |  |  |
| **Day 1 (pre-vaccination)** | N=10 | N=9 | N=8 | N=14 |  | N=3 |  | N=15 |
| % ≥0.5 µg/mL | 40 (12.2, 73.8) | 44 (13.7, 78.8) | 63 (24.5, 91.5) | 64 (35.1, 87.2) |  | 0 (0.0, 70.8) |  | 0 (0.0, 21.8) |
| % ≥1 µg/mL | 30 (6.7, 65.2) | 33 (7.5, 70.1) | 38 (8.5, 75.5) | 36 (12.8, 64.9) |  | 0 (0.0, 70.8) |  | 0 (0.0, 21.8) |
| % ≥2 µg/mL | 0 (0.0, 30.8) | 22 (2.8, 60.0) | 25 (3.2, 65.1) | 21 (4.7, 50.8) |  | 0 (0.0, 70.8) |  | 0 (0.0, 21.8) |
| % ≥3 µg/mL | 0 (0.0, 30.8) | 22 (2.8, 60.0) | 25 (3.2, 65.1) | 21 (4.7, 50.8) |  | 0 (0.0, 70.8) |  | 0 (0.0, 21.8) |
| % ≥5 µg/mL | 0 (0.0, 30.8) | 22 (2.8, 60.0) | 13 (0.3, 52.7) | 14 (1.8, 42.8) |  | 0 (0.0, 70.8) |  | 0 (0.0, 21.8) |
| % ≥8 µg/mL | 0 (0.0, 30.8) | 22 (2.8, 60.0) | 13 (0.3, 52.7) | 0 (0.0, 23.2) |  | 0 (0.0, 70.8) |  | 0 (0.0, 21.8) |
| **30 days post-vaccination** | N=10 | N=9 | N=8 | N=14 |  | N=3 |  | N=15 |
| % ≥0.5 µg/mL | 100 (69.2, 100) | 100 (66.4, 100) | 100 (63.1, 100) | 100 (76.8, 100) |  | 67 (9.4, 99.2) |  | 47 (21.3, 73.4) |
| % ≥1 µg/mL | 100 (69.2, 100) | 100 (66.4, 100) | 100 (63.1, 100) | 100 (76.8, 100) |  | 67 (9.4, 99.2) |  | 27 (7.8, 55.1) |
| % ≥2 µg/mL | 100 (69.2, 100) | 100 (66.4, 100) | 100 (63.1, 100) | 100 (76.8, 100) |  | 33 (0.8, 90.6) |  | 27 (7.8, 55.1) |
| % ≥3 µg/mL | 100 (69.2, 100) | 89 (51.8, 99.7) | 100 (63.1, 100) | 100 (76.8, 100) |  | 33 (0.8, 90.6) |  | 27 (7.8, 55.1) |
| % ≥5 µg/mL | 90 (55.5, 99.8) | 89 (51.8, 99.7) | 100 (63.1, 100) | 100 (76.8, 100) |  | 33 (0.8, 90.6) |  | 27 (7.8, 55.1) |
| % ≥8 µg/mL | 80 (44.4, 97.5) | 89 (51.8, 99.7) | 100 (63.1, 100) | 93 (66.1, 99.8) |  | 33 (0.8, 90.6) |  | 27 (7.8, 55.1) |
| **60 days post-vaccination** | N=10 | N=9 | N=8 | N=14 |  | N=3 |  | N=15 |
| % ≥0.5 µg/mL | 100 (69.2, 100) | 100 (66.4, 100) | 100 (63.1, 100) | 100 (76.8, 100) |  | 67 (9.4, 99.2) |  | 60 (32.3, 83.7) |
| % ≥1 µg/mL | 100 (69.2, 100) | 100 (66.4, 100) | 100 (63.1, 100) | 100 (76.8, 100) |  | 33 (0.8, 90.6) |  | 53 (26.6, 78.7) |
| % ≥2 µg/mL | 100 (69.2, 100) | 100 (66.4, 100) | 100 (63.1, 100) | 100 (76.8, 100) |  | 33 (0.8, 90.6) |  | 33 (11.8, 61.1) |
| % ≥3 µg/mL | 100 (69.2, 100) | 89 (51.8, 99.7) | 100 (63.1, 100) | 100 (76.8, 100) |  | 33 (0.8, 90.6) |  | 33 (11.8, 61.1) |
| % ≥5 µg/mL | 80 (44.4, 97.5) | 89 (51.8, 99.7) | 100 (63.1, 100) | 100 (76.8, 100) |  | 33 (0.8, 90.6) |  | 27 (7.8, 55.1) |
| % ≥8 µg/mL | 80 (44.4, 97.5) | 89 (51.8, 99.7) | 100 (63.1, 100) | 93 (66.1, 99.8) |  | 0 (0.0, 70.8) |  | 20 (4.3, 48.1) |
| ***Serotype III*** |  |  |  |  |  |  |  |  |
| **All** |  |  |  |  |  |  |  |  |
| **Day 1 (pre-vaccination)** | N=14 | N=14 | N=10 | N=15 |  | N=6 |  | N=20 |
| % ≥0.5 µg/mL | 86 (57.2, 98.2) | 64 (35.1, 87.2) | 100 (69.2, 100) | 67 (38.4, 88.2) |  | 0 (0.0, 45.9) |  | 20 (5.7, 43.7) |
| % ≥1 µg/mL | 71 (41.9, 91.6) | 50 (23.0, 77.0) | 100 (69.2, 100) | 47 (21.3, 73.4) |  | 0 (0.0, 45.9) |  | 15 (3.2, 37.9) |
| % ≥2 µg/mL | 71 (41.9, 91.6) | 36 (12.8, 64.9) | 70 (34.8, 93.3) | 47 (21.3, 73.4) |  | 0 (0.0, 45.9) |  | 10 (1.2, 31.7) |
| % ≥3 µg/mL | 71 (41.9, 91.6) | 29 (8.4, 58.1) | 60 (26.2, 87.8) | 27 (7.8, 55.1) |  | 0 (0.0, 45.9) |  | 10 (1.2, 31.7) |
| % ≥5 µg/mL | 50 (23.0, 77.0) | 29 (8.4, 58.1) | 40 (12.2, 73.8) | 20 (4.3, 48.1) |  | 0 (0.0, 45.9) |  | 10 (1.2, 31.7) |
| % ≥8 µg/mL | 43 (17.7, 71.1) | 29 (8.4, 58.1) | 20 (2.5, 55.6) | 20 (4.3, 48.1) |  | 0 (0.0, 45.9) |  | 5 (0.1, 24.9) |
| **30 days post-vaccination** | N=13 | N=13 | N=10 | N=15 |  | N=6 |  | N=20 |
| % ≥0.5 µg/mL | 100 (75.3, 100) | 100 (75.3, 100) | 100 (69.2, 100) | 100 (78.2, 100) |  | 67 (22.3, 95.7) |  | 70 (45.7, 88.1) |
| % ≥1 µg/mL | 100 (75.3, 100) | 100 (75.3, 100) | 100 (69.2, 100) | 100 (78.2, 100) |  | 50 (11.8, 88.2) |  | 65 (40.8, 84.6) |
| % ≥2 µg/mL | 100 (75.3, 100) | 100 (75.3, 100) | 100 (69.2, 100) | 100 (78.2, 100) |  | 50 (11.8, 88.2) |  | 60 (36.1, 80.9) |
| % ≥3 µg/mL | 100 (75.3, 100) | 100 (75.3, 100) | 100 (69.2, 100) | 100 (78.2, 100) |  | 33 (4.3, 77.7) |  | 55 (31.5, 76.9) |
| % ≥5 µg/mL | 100 (75.3, 100) | 100 (75.3, 100) | 100 (69.2, 100) | 100 (78.2, 100) |  | 33 (4.3, 77.7) |  | 55 (31.5, 76.9) |
| % ≥8 µg/mL | 100 (75.3, 100) | 92 (64.0, 99.8) | 100 (69.2, 100) | 100 (78.2, 100) |  | 33 (4.3, 77.7) |  | 50 (27.2, 72.8) |
| **60 days post-vaccination** | N=13 | N=13 | N=10 | N=15 |  | N=5 |  | N=20 |
| % ≥0.5 µg/mL | 100 (75.3, 100) | 100 (75.3, 100) | 100 (69.2, 100) | 100 (78.2, 100) |  | 80 (28.4, 99.5) |  | 80 (56.3, 94.3) |
| % ≥1 µg/mL | 100 (75.3, 100) | 100 (75.3, 100) | 100 (69.2, 100) | 100 (78.2, 100) |  | 40 (5.3, 85.3) |  | 75 (50.9, 91.3) |
| % ≥2 µg/mL | 100 (75.3, 100) | 100 (75.3, 100) | 100 (69.2, 100) | 100 (78.2, 100) |  | 40 (5.3, 85.3) |  | 65 (40.8, 84.6) |
| % ≥3 µg/mL | 100 (75.3, 100) | 100 (75.3, 100) | 100 (69.2, 100) | 100 (78.2, 100) |  | 40 (5.3, 85.3) |  | 65 (40.8, 84.6) |
| % ≥5 µg/mL | 100 (75.3, 100) | 100 (75.3, 100) | 100 (69.2, 100) | 100 (78.2, 100) |  | 40 (5.3, 85.3) |  | 50 (27.2, 72.8) |
| % ≥8 µg/mL | 100 (75.3, 100) | 92 (64.0, 99.8) | 100 (69.2, 100) | 100 (78.2, 100) |  | 40 (5.3, 85.3) |  | 50 (27.2, 72.8) |
| **<LLQ** |  |  |  |  |  |  |  |  |
| **Day 1 (pre-vaccination)** | N=10 | N=13 | N=9 | N=11 |  | N=4 |  | N=15 |
| % ≥0.5 µg/mL | 80 (44.4, 97.5) | 62 (31.6, 86.1) | 100 (66.4, 100) | 55 (23.4, 83.3) |  | 0 (0.0, 60.2) |  | 0 (0.0, 21.8) |
| % ≥1 µg/mL | 60 (26.2, 87.8) | 46 (19.2, 74.9) | 100 (66.4, 100) | 27 (6.0, 61.0) |  | 0 (0.0, 60.2) |  | 0 (0.0, 21.8) |
| % ≥2 µg/mL | 60 (26.2, 87.8) | 31 (9.1, 61.4) | 67 (29.9, 92.5) | 27 (6.0, 61.0) |  | 0 (0.0, 60.2) |  | 0 (0.0, 21.8) |
| % ≥3 µg/mL | 60 (26.2, 87.8) | 23 (5.0, 53.8) | 56 (21.2, 86.3) | 18 (2.3, 51.8) |  | 0 (0.0, 60.2) |  | 0 (0.0, 21.8) |
| % ≥5 µg/mL | 30 (6.7, 65.2) | 23 (5.0, 53.8) | 33 (7.5, 70.1) | 9 (0.2, 41.3) |  | 0 (0.0, 60.2) |  | 0 (0.0, 21.8) |
| % ≥8 µg/mL | 30 (6.7, 65.2) | 23 (5.0, 53.8) | 22 (2.8, 60.0) | 9 (0.2, 41.3) |  | 0 (0.0, 60.2) |  | 0 (0.0, 21.8) |
| **30 days post-vaccination** | N=10 | N=12 | N=9 | N=11 |  | N=4 |  | N=15 |
| % ≥0.5 µg/mL | 100 (69.2, 100) | 100 (73.5, 100) | 100 (66.4, 100) | 100 (71.5, 100) |  | 75 (19.4, 99.4) |  | 60 (32.3, 83.7) |
| % ≥1 µg/mL | 100 (69.2, 100) | 100 (73.5, 100) | 100 (66.4, 100) | 100 (71.5, 100) |  | 50 (6.8, 93.2) |  | 53 (26.6, 78.7) |
| % ≥2 µg/mL | 100 (69.2, 100) | 100 (73.5, 100) | 100 (66.4, 100) | 100 (71.5, 100) |  | 50 (6.8, 93.2) |  | 53 (26.6, 78.7) |
| % ≥3 µg/mL | 100 (69.2, 100) | 100 (73.5, 100) | 100 (66.4, 100) | 100 (71.5, 100) |  | 25 (0.6, 80.6) |  | 47 (21.3, 73.4) |
| % ≥5 µg/mL | 100 (69.2, 100) | 100 (73.5, 100) | 100 (66.4, 100) | 100 (71.5, 100) |  | 25 (0.6, 80.6) |  | 47 (21.3, 73.4) |
| % ≥8 µg/mL | 100 (69.2, 100) | 92 (61.5, 99.8) | 100 (66.4, 100) | 100 (71.5, 100) |  | 25 (0.6, 80.6) |  | 40 (16.3, 67.7) |
| **60 days post-vaccination** | N=10 | N=12 | N=9 | N=11 |  | N=4 |  | N=15 |
| % ≥0.5 µg/mL | 100 (69.2, 100) | 100 (73.5, 100) | 100 (66.4, 100) | 100 (71.5, 100) |  | 75 (19.4, 99.4) |  | 73 (44.9, 92.2) |
| % ≥1 µg/mL | 100 (69.2, 100) | 100 (73.5, 100) | 100 (66.4, 100) | 100 (71.5, 100) |  | 25 (0.6, 80.6) |  | 67 (38.4, 88.2) |
| % ≥2 µg/mL | 100 (69.2, 100) | 100 (73.5, 100) | 100 (66.4, 100) | 100 (71.5, 100) |  | 25 (0.6, 80.6) |  | 53 (26.6, 78.7) |
| % ≥3 µg/mL | 100 (69.2, 100) | 100 (73.5, 100) | 100 (66.4, 100) | 100 (71.5, 100) |  | 25 (0.6, 80.6) |  | 53 (26.6, 78.7) |
| % ≥5 µg/mL | 100 (69.2, 100) | 100 (73.5, 100) | 100 (66.4, 100) | 100 (71.5, 100) |  | 25 (0.6, 80.6) |  | 40 (16.3, 67.7) |
| % ≥8 µg/mL | 100 (69.2, 100) | 92 (61.5, 99.8) | 100 (66.4, 100) | 100 (71.5, 100) |  | 25 (0.6, 80.6) |  | 40 (16.3, 67.7) |

Abbreviations: GBS, Group B streptococcus; No Adj, non-adjuvanted formulation; Alum, aluminum hydroxide-adjuvanted formulation; MF59 full, full-dosage MF59-adjuvanted formulation; MF59 half, half-dosage MF59-adjuvanted formulation; LLQ, lower limit of quantitation; N, number of women with available results in each group; NA, not applicable.

Analyses on all women regardless of their baseline LLQ status (“All”) and on women with baseline serotype-specific anti-GBS antibody concentrations below the LLQs (“<LLQ”). Baseline refers to the pre-vaccination time point in the parent study for the prior GBS and prior placebo groups and to the pre-vaccination time point (day 1) in the extension study for the naïve group. Day 1 refers to the pre-vaccination time point in the extension study.

**Supplementary** **Table 2. Serotype-specific geometric mean antibody concentrations (with 95% confidence intervals) at different time points, and geometric mean ratios at day 31 or day 61 compared to baseline or day 1 (per protocol immunogenicity set)**

|  | | **Prior GBS** | | | | **Combined Prior GBS** | | | **Prior placebo** | **Naïve** | **No Prior GBS** |
| --- | --- | --- | --- | --- | --- | --- | --- | --- | --- | --- | --- |
| **Parent study vaccine:** | | **GBS No Adj** | **GBS Alum** | **GBS MF59 Full** | **GBS MF59 Half** | | **GBS No Adj/alum** | **GBS MF59** | **Placebo** | **NA** | **Placebo/NA** |
| **Extension study vaccine:** | | **GBS No Adj** | **GBS No Adj** | **GBS No Adj** | **GBS No Adj** | | **GBS No Adj** | **GBS No Adj** | **GBS No Adj** | **GBS No Adj** | **GBS No Adj** |
| ***Serotype Ia*** | | N=14 | N=14 | N=9 | N=15 | | N=28 | N=24 | N=6 | N=20 | N=26 |
| GMC | Baseline | 1.17  (0.31, 4.37) | 0.37  (0.10, 1.39) | 0.43  (0.09, 2.26) | 1.00  (0.28, 3.57) | | 0.66  (0.26, 1.66) | 0.73  (0.27, 1.99) | 0.52  (0.04, 6.14) | NA | NA |
| GMC | Day 1 | 18.12  (5.00, 66) | 12.26  (3.39, 44) | 6.87  (1.38, 34) | 6.95  (2.00, 24) | | 14.91  (6.05, 37) | 6.92  (2.61, 18) | 1.33  (0.19, 9.48) | 0.33  (0.11, 0.98) | 0.46  (0.18, 1.17) |
| GMC | Day 31 | 142.39  (54, 379) | 271.76  (107, 693) | 336.65  (105, 1079) | 151.48  (61, 375) | | 199.53  (100, 397) | 205.10  (99, 426) | 141.90  (25, 814) | 18.72  (8.53, 41) | 26.07  (13, 54) |
| GMC | Day 61 | 108.81  (48, 246) | 218.67  (100, 477) | 254.28  (96, 672) | 116.77  (55, 249) | | 156.66  (88, 279) | 156.97  (85, 290) | 130.06  (30, 558) | 23.77  (12, 46) | 31.34  (17, 58) |
| GMR | Day 31 : Baseline | 148.92  (39, 566) | 638.75  (177, 2311) | 711.55  (143, 3539) | 183.59  (53, 636) | | 316.85  (123, 814) | 305.13  (112, 831) | 266.53  (24, 2956) | 47.35  (16, 139) | 63.15  (23, 172) |
| GMR | Day 61 : Baseline | 114.20  (34, 387) | 513.03  (158, 1663) | 536.85  (124, 2327) | 141.88  (46, 442) | | 248.88  (105, 592) | 233.69  (93, 586) | 244.20  (27, 2203) | 60.01  (22, 160) | 75.83  (30, 190) |
| GMR | Day 31 : Day 1 | 9.94  (2.94, 34) | 19.34  (5.98, 63) | 45.06  (10, 195) | 26.32  (8.46, 82) | | 14.04  (6.10, 32) | 32.20  (13, 78) | 35.54  (5.91, 214) | 47.35  (18, 126) | 44.32  (19, 104) |
| GMR | Day 61 : Day 1 | 7.62  (2.60, 22) | 15.53  (5.52, 44) | 34.00  (9.35, 124) | 20.34  (7.48, 55) | | 11.03  (5.26, 23) | 24.66  (11, 54) | 27.09  (4.79, 153) | 60.01  (25, 143) | 51.19  (24, 110) |
| ***Serotype Ib*** | | N=14 | N=14 | N=10 | N=15 | | N=28 | N=25 | N=6 | N=20 | N=26 |
| GMC | Baseline | 0.19  (0.09, 0.41) | 0.15  (0.07, 0.32) | 0.16  (0.07, 0.39) | 0.10  (0.05, 0.20) | | 0.17  (0.10, 0.29) | 0.12  (0.07, 021) | 0.14  (0.04, 0.55) | NA | NA |
| GMC | Day 1 | 1.34  (0.44, 4.10) | 1.87  (0.61, 5.70) | 1.90  (0.51, 7.12) | 0.87  (0.30, 2.55) | | 1.58  (0.73, 3.45) | 1.19  (0.52, 2.71) | 0.16  (0.03, 0.86) | 0.17  (0.07, 0.44) | 0.17  (0.08, 0.38) |
| GMC | Day 31 | 80.62  (28, 232) | 74.96  (27, 207) | 85.90  (26, 286) | 89.59  (33, 241) | | 77.65  (38, 159) | 88.02  (42, 186) | 6.61  (0.99, 44) | 3.55  (1.52, 8.34) | 3.94  (1.84, 8.46) |
| GMC | Day 61 | 56.32  (22, 145) | 57.82  (23, 143) | 65.53  (22, 192) | 59.13  (24, 143) | | 57.09  (30, 108) | 61.62  (32, 120) | 5.51  (1.01, 30) | 4.47  (2.09, 9.58) | 4.63  (2.35, 9.14) |
| GMR | Day 31 : Baseline | 547.17  (192, 1559) | 509.34  (186, 1397) | 582.97  (177, 1924) | 613.97  (232, 1628) | | 527.22  (258, 1076) | 601.38  (287, 1262) | 44.99  (6.81, 297) | 24.09  (10, 56) | 26.74  (13, 57) |
| GMR | Day 61 : Baseline | 381.69  (150, 974) | 392.60  (159, 968) | 444.01  (153, 1292) | 407.73  (171, 975) | | 387.31  (205, 732) | 421.87  (218, 817) | 37.55  (6.94, 203) | 30.24  (14, 64) | 31.35  (16, 62) |
| GMR | Day 31 : Day 1 | 81.16  (25, 259) | 41.74  (14, 128) | 49.76  (13, 187) | 70.52  (24, 207) | | 57.49  (26, 127) | 61.34  (27, 140) | 36.66  (6.66, 202) | 24.09  (9.46, 61) | 26.54  (12, 60) |
| GMR | Day 61 : Day 1 | 56.61  (20, 163) | 32.17  (12, 89) | 37.90  (11, 127) | 46.83  (17, 125) | | 42.23  (21, 87) | 43.03  (20, 91) | 29.91  (5.43, 165) | 30.24  (13, 71) | 30.18  (14, 64) |
| ***Serotype III*** | | N=14 | N=13 | N=10 | N=15 | | N=27 | N=25 | N=6 | N=20 | N=26 |
| GMC | Baseline | 0.41  (0.20, 0.82) | 0.17  (0.09, 0.36) | 0.17  (0.08, 0.39) | 0.25  (0.13, 0.49) | | 0.27  (0.16, 0.45) | 0.21  (0.13, 0.36) | 0.15  (0.04, 0.54) | NA | NA |
| GMC | Day 1 | 6.10  (2.44, 15) | 1.68  (0.65, 4.34) | 3.91  (1.32, 12) | 1.59  (0.65, 3.84) | | 3.28  (1.67, 6.43) | 2.27  (1.13, 4.58) | 0.15  (0.04, 0.59) | 0.31  (0.14, 0.66) | 0.26  (0.13, 0.51) |
| GMC | Day 31 | 111.30  (42, 294) | 104.55  (40, 277) | 216.39  (71, 655) | 109.23  (44, 269) | | 107.88  (55, 212) | 143.64  (72, 286) | 2.66  (0.46, 15) | 5.33  (2.43, 12) | 4.74  (2.34, 9.60) |
| GMC | Day 61 | 91.30  (39, 214) | 80.94  (34, 190) | 200.78  (76, 531) | 81.71  (37, 180) | | 85.97  (47, 157) | 117.11  (64, 126) | 2.67  (0.57, 12) | 5.82  (2.92, 12) | 5.11  (2.74, 9.54) |
| GMR | Day 31 : Baseline | 435.31  (163, 1162) | 484.50  (182, 1293) | 1006.55  (329, 3082) | 454.14  (182, 1132) | | 459.25  (231, 912) | 624.38  (310, 1257) | 13.00  (2.22, 76) | 20.80  (9.43, 46) | 19.23  (9.42, 39) |
| GMR | Day 61 : Baseline | 355.85  (149, 848) | 376.67  (158, 898) | 938.09  (348, 2526) | 339.47  (151, 762) | | 366.12  (198, 676) | 509.77  (273, 953) | 13.14  (2.74, 63) | 22.64  (11, 46) | 20.67  (11, 39) |
| GMR | Day 31 : Day 1 | 25.04  (8.68, 72) | 50.38  (17, 145) | 44.36  (13, 148) | 71.29  (27, 191) | | 35.52  (17, 75) | 58.97  (28, 126) | 11.90  (2.50, 57) | 20.80  (8.85, 49) | 18.28  (8.71, 38) |
| GMR | Day 61 : Day 1 | 20.47  (8.14, 51) | 39.17  (16, 98) | 41.34  (14, 118) | 53.29  (23, 126) | | 28.32  (15, 54) | 48.14  (25, 93) | 20.65  (4.67, 91) | 22.64  (11, 48) | 22.23  (12, 43) |

Abbreviations: GBS, Group B streptococcus; No Adj, non-adjuvanted formulation; Alum, aluminum hydroxide-adjuvanted formulation; MF59 full, full-dosage MF59-adjuvanted formulation; MF59 half, half-dosage MF59-adjuvanted formulation; N, number of women with available results in each group (varies slightly per time point); GMC, adjusted geometric mean concentration; GMR, geometric mean ratio; NA, not applicable.

Analyses on all women regardless of their baseline lower limit of quantitation status. Baseline refers to the pre-vaccination time point in the parent study for the prior GBS and prior placebo groups and to the pre-vaccination time point (day 1) in the extension study for the naïve group. Day 1 refers to the pre-vaccination time point in the extension study. For the naïve group, as baseline and day 1 refer to the same time point, we presented the baseline GMCs as “NA”. Antibody concentrations <LLQ were given an arbitrary value of half the LLQ. Baseline and day 1 GMCs, and GMRs were calculated using an ANOVA model, the other GMCs using an ANCOVA model with log_10_ pre-vaccination concentrations as covariate.

**Supplementary Table 3. Serotype-specific adjusted geometric mean antibody concentrations (with 95% confidence intervals) at different time points, based on baseline antibody concentration (per protocol immunogenicity set)**

|  | **Prior GBS** | | | |  | **Prior placebo** |  | **Naïve** |
| --- | --- | --- | --- | --- | --- | --- | --- | --- |
| **Parent study vaccine:** | **GBS No Adj** | **GBS Alum** | **GBS MF59 Full** | **GBS MF59 Half** |  | **Placebo** |  | **NA** |
| **Extension study vaccine:** | **GBS No Adj** | **GBS No Adj** | **GBS No Adj** | **GBS No Adj** |  | **GBS No Adj** |  | **GBS No Adj** |
| ***Serotype Ia*** |  |  |  |  |  |  |  |  |
| **<LLQ** | N=7 | N=10 | N=7 | N=9 |  | N=2 |  | N=13 |
| Baseline | 0.12 (0.12, 0.12) | 0.12 (0.12, 0.12) | 0.12 (0.12, 0.12) | 0.12 (0.12, 0.12) |  | 0.12 (0.12, 0.12) |  | NA |
| Day 1 | 2.57 (0.69, 9.58) | 4.14 (1.38, 12) | 2.88 (0.77, 11) | 1.55 (0.49, 4.93) |  | 1.60 (0.14, 19) |  | 0.12 (0.04, 0.31) |
| Day 31 | 93.31 (23, 378) | 176.47 (55, 569) | 267.59 (66, 1084) | 111.49 (32, 383) |  | 34.44 (2.51, 472) |  | 8.39 (3.00, 23) |
| Day 61 | 61.26 (19, 193) | 137.83 (53, 360) | 194.06 (62, 610) | 77.72 (28, 214) |  | 37.34 (4.38, 319) |  | 12.56 (5.42, 29) |
| **≥LLQ** | N=7 | N=4 | N=2 | N=6 |  | N=2 |  | N=7 |
| Baseline | 11.67 (2.19, 62) | 6.73 (0.73, 62) | 43.71 (1.90, 1003) | 24.92 (4.08, 152) |  | 2.31 (0.10, 53) |  | NA |
| Day 1 | 127.69 (34, 483) | 185.63 (32, 1079) | 143.08 (12, 1723) | 66.18 (16, 278) |  | 12.56 (1.04, 151) |  | 2.33 (0.62, 8.83) |
| Day 31 | 287.44 (68, 1211) | 575.20 (102, 3252) | 247.25 (20, 3043) | 240.59 (56, 1034) |  | 1063.83 (88, 12915) |  | 96.77 (24, 392) |
| Day 61 | 273.21 (75, 994) | 488.57 (103, 2314) | 229.56 (24, 2186) | 226.82 (61, 840) |  | 774.59 (82, 7286) |  | 84.76 (24, 298) |
| ***Serotype Ib*** |  |  |  |  |  |  |  |  |
| **<LLQ** | N=10 | N=9 | N=8 | N=14 |  | N=3 |  | N=15 |
| Baseline | 0.08 (0.08, 0.08) | 0.08 (0.08, 0.08) | 0.08 (0.08, 0.08) | 0.08 (0.08, 0.08) |  | 0.08 (0.08, 0.08) |  | NA |
| Day 1 | 0.36 (0.15, 0.86) | 0.53 (0.21, 1.35) | 0.74 (0.28, 1.99) | 0.65 (0.31, 1.38) |  | 0.08 (0.02, 0.39) |  | 0.08 (0.04, 0.16) |
| Day 31 | 60.78 (20, 182) | 69.05 (22, 220) | 65.50 (19, 224) | 53.01 (21, 134) |  | 1.47 (0.20, 11) |  | 0.79 (0.32, 1.95) |
| Day 61 | 40.59 (15, 111) | 47.71 (16, 138) | 47.58 (15, 147) | 34.12 (15, 80) |  | 1.32 (0.21, 8.37) |  | 1.19 (0.52, 2.71) |
| **≥LLQ** | N=4 | N=5 | N=2 | N=1 |  | N=1 |  | N=5 |
| Baseline | 1.96 (0.56, 6.85) | 0.52 (0.17, 1.60) | 3.12 (0.53, 18) | 3.42 (0.28, 42) |  | 0.82 (0.07, 10) |  | NA |
| Day 1 | 37.24 (14, 100) | 17.96 (7.45, 43) | 81.90 (20, 329) | 47.48 (6.63, 340) |  | 5.39 (0.75, 39) |  | 1.92 (0.80, 4.63) |
| Day 31 | 287.34 (90, 917) | 121.26 (44, 333) | 338.80 (78, 1469) | 373.02 (48, 2877) |  | 557.46 (75, 4142) |  | 557.03 (225, 1379) |
| Day 61 | 228.26 (72, 726) | 118.94 (43, 326) | 300.10 (69, 1296) | 367.26 (48, 2818) |  | 378.70 (51, 2800) |  | 403.16 (163, 996) |
| ***Serotype III*** |  |  |  |  |  |  |  |  |
| **<LLQ** | N=10 | N=12 | N=9 | N=11 |  | N=4 |  | N=15 |
| Baseline | 0.15 (0.15, 0.15) | 0.15 (0.15, 0.15) | 0.15 (0.15, 0.15) | 0.15 (0.15, 0.15) |  | 0.15 (0.15, 0.15) |  | NA |
| Day 1 | 2.45 (1.14, 5.27) | 1.21 (0.60, 2.44) | 3.65 (1.62, 8.19) | 0.85 (0.41, 1.77) |  | 0.15 (0.04, 0.49) |  | 0.15 (0.08, 0.27) |
| Day 31 | 109.31 (40, 301) | 77.97 (31, 197) | 188.63 (65, 549) | 126.89 (48, 333) |  | 1.90 (0.38, 9.44) |  | 2.12 (0.93, 4.84) |
| Day 61 | 83.19 (33, 208) | 60.01 (26, 138) | 169.45 (65, 444) | 87.40 (37, 209) |  | 1.92 (0.45, 8.16) |  | 2.52 (1.20, 5.32) |
| **≥LLQ** | N=4 | N=1 | N=1 | N=4 |  | N=0 |  | N=5 |
| Baseline | 5.33 (0.76, 37) | 1.41 (0.03, 68) | 0.74 (0.02, 36) | 1.07 (0.15, 7.46) |  | NA |  | NA |
| Day 1 | 59.80 (6.82, 525) | 82.22 (1.07, 6326) | 7.25 (0.09, 558) | 8.75 (1.00, 77) |  | NA |  | 2.79 (0.40, 19) |
| Day 31 | 171.67 (13, 2214) | 270.01 (3.46, 21064) | 149.93 (1.81, 12454) | 120.00 (13, 1113) |  | NA |  | 136.68 (19, 979) |
| Day 61 | 186.63 (24, 1439) | 231.17 (7.12, 7509) | 180.29 (5.28, 6158) | 107.21 (18, 635) |  | NA |  | 116.43(24, 561) |

Abbreviations: GBS, Group B streptococcus; No Adj, non-adjuvanted formulation; Alum, aluminum hydroxide-adjuvanted formulation; MF59 full, full-dosage MF59-adjuvanted formulation; MF59 half, half-dosage MF59-adjuvanted formulation; LLQ, lower limit of quantitation; N, number of women with available results in each group; NA, not applicable.

Analyses on women with baseline serotype-specific anti-GBS antibody concentrations below the LLQs (“<LLQ”) or greater than/equal to the LLQs (“≥LLQ”). Baseline refers to the pre-vaccination time point in the parent study for the prior GBS and prior placebo groups and to the pre-vaccination time point (day 1) in the extension study for the naïve group. Day 1 refers to the pre-vaccination time point in the extension study. For the naïve group, as baseline and day 1 refer to the same time point, we presented the baseline GMCs as “NA”. Antibody concentrations <LLQ were given an arbitrary value of half the LLQ. Baseline and day 1 geometric mean concentrations were calculated using an ANOVA model, the other geometric mean concentrations using an ANCOVA model with log_10_ pre-vaccination concentrations as covariate.

**Supplementary Figures**

**Supplementary Figure 1. Reverse cumulative distribution curves of serotype-specific anti-GBS antibody concentrations pre-vaccination (per protocol immunogenicity set)**


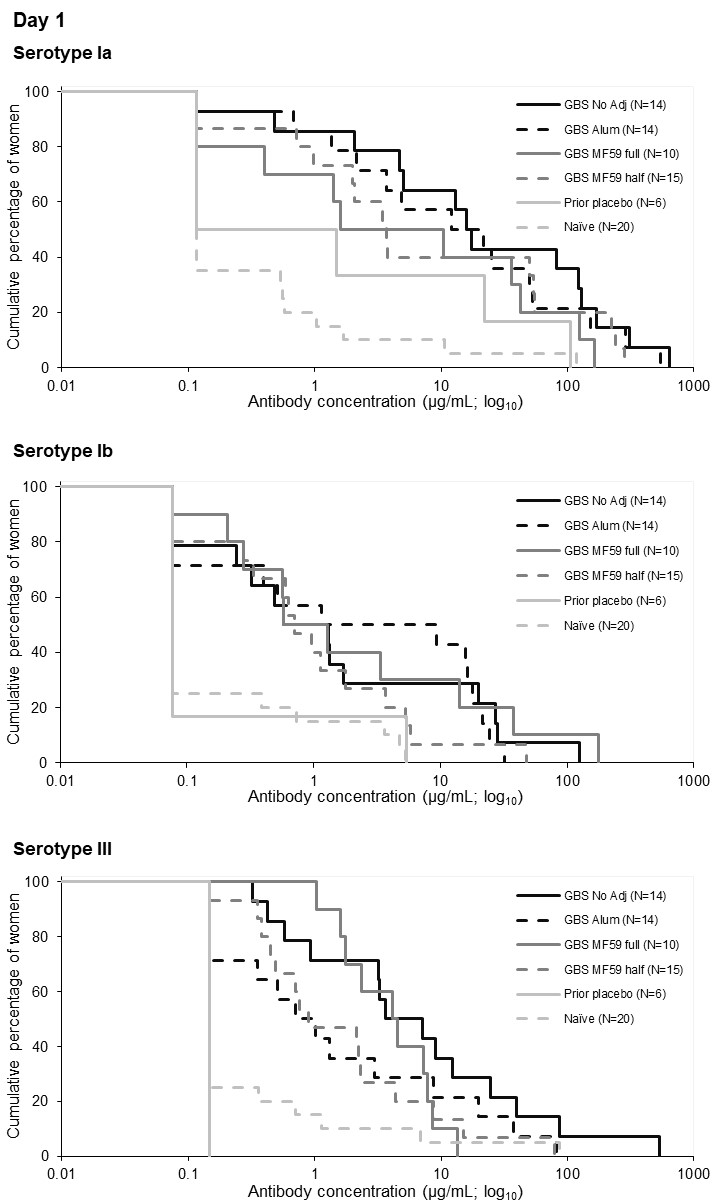


Abbreviations: GBS, Group B streptococcus; No Adj, non-adjuvanted formulation; Alum, aluminum hydroxide-adjuvanted formulation; MF59 full, full-dosage MF59-adjuvanted formulation; MF59 half, half-dosage MF59-adjuvanted formulation; N, number of women with available results in each group.

Analyses on all women regardless of their baseline lower limit of quantitation status. Day 1 refers to the pre-vaccination time point in the extension study.
